# Are study strategies related to medical licensing exam performance?

**DOI:** 10.5116/ijme.5439.6491

**Published:** 2014-11-02

**Authors:** Courtney West, Terri Kurz, Sherry Smith, Lori Graham

**Affiliations:** 1 Office of Medical Education, Department of Internal Medicine, Texas A&M University, Health Science Center, College of Medicine, USA; 2 Office of Medical Education, Texas A&M University, Health Science Center, College of Medicine, USA

**Keywords:** Study strategies, medical licensing exams, academic performance

## Abstract

**Objectives:**

To examine the relationship between study strategies and performance on a high stakes medical licensing exam entitled the United States Medical Licensing Examination Step 1.

**Methods:**

The action research project included seventy nine student participants at the Texas A&M Health Science Center College of Medicine during their pre-clinical education. Data collection included pre-matriculation and matriculation academic performance data, standardized exam data, and the Learning and Study Strategies Instrument. Multiple regression analyses were conducted. For both models, the dependent variable was the Step 1 score, and the independent variables included Medical College Admission Test, Undergraduate Grade Point Average, Year 1 Average, Year 2 Average, Customized National Board of Medical Examiners Average, Comprehensive Basic Science Exam score, and Learning and Study Strategy Instrument sub-scores. Model 2 added Comprehensive Basic Science Self-Assessment average.

**Results:**

Concentration (Model 1 - β = .264; Model 2 - β = .254) was the only study strategy correlated with Step 1 performance. The other statistically significant predictors were Customized National Board of Medical Examiners Average (β = .315) and Year 2 Average (β = .280) in Model 1 and Comprehensive Basic Science Self-Assessment Average (β = .338) in Model 2.

**Conclusions:**

There does appear to be a relationship between the study strategy concentration and Step 1 licensing exam performance. Teaching students to practice and utilize certain techniques to improve concentration skills when preparing for and taking exams may help improve licensing exam scores.

## Introduction

High stakes exams are the norm in all facets of education including medical education. In the United States, the number of Graduate Medical Education (GME) slots have not increased, and residencies appear to be using licensing exam scores, such as the United States Medical Licensing Examination (USMLE), to screen applicants.^1,2^ Therefore, the stakes for these exams have risen exponentially, and high licensing exam scores are not only coveted but essential. This is certainly the case for International Medical Graduates (IMGs) who are competing for GME positions in the United States.^3^ Since the USMLE Step 1 exam is the initial licensing exam in the United States which assesses students’ application of basic science knowledge to clinical scenarios presented as clinical vignettes, numerous studies have focused on identifying possible performance predictors. Pre-matriculation and matriculation data have been investigated in detail, and medical gross anatomy performance,^4^ medical school performance,^5^ and prior standardized test performance^6,7^ appear to be related to Step 1 performance. 

While academic performance in medical school and previous standardized test performance appear to be predictors of USMLE Step 1 performance, the ability of the student to sufficiently prepare for and perform well on exam day are key elements. For instance, developing a sound study schedule^5^ and completing thousands of q-bank questions during the preparation phase appear to be associated with Step 1 success. Students who have the ability to self-regulate and monitor their learning are more effective learners due to their repertoire of learning and study strategies.^8^ Not all students enter medical school and/or other higher education institutions with the same internal collection of learning and study strategies and, as a result, some will experience academic difficulties.^9^ Accordingly, some institutions invest time and resources in an effort to facilitate strategic learning where higher-order thinking and interpretation mechanisms are required.^10,11^ Many of these programs also integrate study strategies such as test taking, note taking, and ways to improve memory and concentration into the curriculum. This focused instruction provides students with opportunities to become more efficient at self-regulated learning. These self-regulated learners then know how to adjust their learning and study strategies to address various learning situations and utilize certain skills which lead to their academic success.

Research devoted to the improvement of learning yielded the Strategic Learning Model.^12^ This model is built upon the premise that successful learners exemplify certain skills, opinions, behaviors, and thought patterns and is the basis for the Learning and Study Strategies Inventory (LASSI).^12^ The LASSI is a widely utilized instrument for assessing aspects of self-regulated learning in higher education by evaluating students’ learning and study strategies. The LASSI subtests have been shown to be generalizable to different populations in higher education, including undergraduates,^13^ pharmacy students,^14^ chiropractic students,^15^ and medical students.^16,17^

Studies have been conducted to explore the relationship between learning and study strategies and academic performance of health care students^15-17^ utilizing the LASSI. These studies have found correlations between learning and study strategies and first semester academic performance and first year Grade Point Average (GPA). For example, West and Sadoski^17^ found that time management and self-testing were strong predictors of first-semester academic performance. Sleight and Mavis^16^ found that students that scored at the top rank of Medical College Admission Test (MCAT) performance scored higher on motivation and concentration than their peers with low and medium scores. These studies suggest that certain learning and study strategies are consistently related to achievement. 

While these studies have shown correlations between learning and study strategies and academic performance, no studies to date have explored the correlation between learning and study strategies and performance on the USMLE Step 1 exam. However, a study has been conducted examining the relationship between learning and study strategies and performance on the National Board of Chiropractic Examiners (NBCE) licensure examination.^18^ This research found LASSI subtests of anxiety, concentration, selecting main ideas and test strategies to be significant predictors of NBCE scores. Understanding the relationship between learning and study strategies and performance on high stakes exams can inform exam preparation and performance, educational intervention, and curriculum planning. The aim of our study is to examine the relationship between study strategies and performance on a high stakes medical licensing exam entitled USMLE Step 1. This investigation is important, because if certain study strategies are related to licensing exam performance, they can be targeted through coaching and other interventions to enhance individuals’ performance on similar high stakes exams. Since prior research suggests that study strategies are related to preclinical coursework performance^8^ in medical school, we hypothesized that there would be a relationship between study strategies and Step 1 performance. Our research question is: Are specific study strategies related to Step 1 licensing exam performance? 

## Methods 

### Study design

This action research project was initiated during medical students’ first year orientation. This study design was selected due to its’ “practicality in terms of context,” our medical school, and the “participatory nature” of the research.^19^ We decided to utilize O’Leary’s research cycle which includes the elements of observing, reflecting, planning, and acting.^20^

### Participants and sampling method 

Convenience sampling was utilized. The participants included pre-clinical medical students in one cohort at the Texas A&M Health Science Center. One hundred and six first year students agreed to participate in the study, signed Texas A&M University Institutional Review Board (IRB) approved consent forms, had complete pre-matriculation data including Medical College Admission Test (MCAT) scores, and took the Learning and Study Strategies Instrument (LASSI) during orientation. Of those, 79 students completed all of the requirements and remained on cycle, submitted National Board of Medical Examiners (NBME) Comprehensive Basic Science Self-Assessment (CBSSA) information, and completed the Step 1 exam at the conclusion of their pre-clinical training which is at the end of their second year of medical school. 

### Data collection 

The data collected in this study included pre-matriculation and matriculation academic performance data, standardized exam data, and the Learning and Study Strategy Inventory (LASSI). The ten LASSI subscale scores were obtained during first year orientation, and pre-matriculation and matriculation academic performance data as well as standardized exam data were collected for each participant at the end of their pre-clinical training. 

### Pre-matriculation academic performance data 

The Medical College Admission Test (MCAT) is a standardized exam which assesses knowledge of scientific concepts, problem solving, critical thinking, and writing. It is divided into verbal reasoning, biological sciences, physical sciences, and writing sample subtests. The total MCAT scaled score (range 3-45) was used in this study. From 2011 to 2013, the MCAT total score mean was 25.2 with a Standard Deviation of 6.4.^21^ Undergraduate Grade Point Average (UGPA) reflects a student’s overall performance in his/her undergraduate studies. The UGPA used in this study ranges from 0.0 to 4.0 and is not restricted to performance in science courses. 

### Matriculation academic performance data 

Year 1 Average is determined by academic performance in our integrated first semester curriculum including Gross Anatomy, Histology, Biochemistry, Genetics, Cell Physiology, and Pharmacology as well as our Introduction to Disease and Neuroscience blocks which take place in the second semester of year 1. 

Year 2 Average is determined by academic performance in our integrated curriculum consisting of Hematology/Oncology, Cardiovascular, Respiratory, Renal/Genitourinary, Gastrointestinal/Nutrition, Endocrinology/Reproductive Science/Human Sexuality, and Integument/Musculoskeletal organ system blocks. 

### Standardized exam data

Customized National Board of Medical Examiners (NBME) Average is an average of three customized basic science exams created by our faculty at the conclusion of each semester which serve as barrier exams. Faculty select questions from a test item bank created by the NBME. The monitored exams are timed to mimic the pacing of the Step 1 exam. The total percent correct is the score utilized. 

The Comprehensive Basic Science Exam (CBSE) is a four hour exam offered by the NBME which consists of deselected Step 1 questions. It is designed to gauge one’s readiness for the Step 1 exam and is required by our institution. This assessment is taken prior to our students 4-6 week intense study period, so it is often viewed as a baseline assessment. 

The Comprehensive Basic Science Self-Assessments (CBSSAs) are four hour exams consisting of deselected Step 1 questions. They are similar to the CBSE but are self-assessments. There are six CBSSAs available from the National Board of Medical Examiners (NBME). Students are encouraged to take these assessments and use them as benchmarks as they focus on Step 1 preparation during their 4-6 week study period. Some students took multiple CBSSAs, so the average score is used in this study. 

The USMLE Step 1 exam is an 8 hour multiple choice exam which is divided into 7 blocks of questions. Each timed question block contains 46 questions that must be completed in one hour. The item stems are typically clinical scenarios which require students to apply basic science concepts. The Step 1 exam is the first of three required licensing exams in the United States.

### Learning and study strategy inventory 

The Learning and Study Strategy Inventory (LASSI) is a norm referenced 80-item assessment which includes statements that students rank on a five-point Likert-type scale. Items measure student awareness about the use of learning and study strategies related to the Strategic Learning Model. The performance profile consists of ten sub-scores (Anxiety, Attitude, Motivation, Concentration, Information Processing, Self-Testing, Selecting Main Idea, Study Aids, Time Management, and Test-taking Strategies) which are related to the three components of strategic learning (Will, Skill, and Self-Regulation). The alpha-reliabilities range from .73-.89, and the test-retest correlation is .88 for the total instrument.^22^

### Data analysis

To investigate how study strategies obtained through the LASSI influence Step I performance, the analysis consisted of descriptive statistics, a correlation matrix, and multiple regression analyses. The assumptions of regression analysis appeared to be satisfied. For example, the review of the data plots indicated that the variables were normally distributed. Scatterplots of residuals identified a linear relationship between the independent and dependent variables, and reliability estimates, Cronbach’s alpha, were calculated.^23^ The dependent variable for the regression models was the Step 1 score. For the first regression model, the independent variables were: MCAT, UGPA, Year 1 Average, Year 2 Average, Customized NBME Average, CBSE score, and the ten LASSI sub-scores: Anxiety, Attitude, Motivation, Concentration, Information Processing, Self-Testing, Selecting Main Idea, Study Aids, Time Management, and Test Taking Strategies. The second regression model included the same independent variables as above but added CBSSA average. 

## Results 

Means, standard deviations, and correlations for the academic data, exam data, and LASSI subscales are in Table 1. The Cronbach’s alpha for the LASSI was .81. 

The Model 1 independent variables MCAT, UGPA, Year I Average, Year 2 Average, Customized NBME Average, and LASSI sub-scores accounted for 65.8% of the variance. Model 2 independent variables (adding CBSSA average) accounted for 71.4% of the variance.

### Regression model 1 

The sixteen independent variables accounted for 65.8% of the variance in Step 1 performance (Table 2). Of the three statistically significant predictors, CBSE (β = .315) was the strongest predictor followed by Year 2 Average (β = .280) and then Concentration (β = .264) (Table 2). 

**Table 1.** Item Means, Standard Deviations, and Inter-item Correlations (n=79) ^[Table-fn fn15890]^

Variables	1	2	3	4	5	6	7	8	9	10	11	12	13	14	15	16	17	18	Mean	SD
Academic Performance Data																				
1. Step 1	-																		224.76	15.04
2. MCAT	.27	-																	29.20	3.24
3. UGPA	.19	.15	-																3.65	.24
4. Year 1 Average	.71	.20	.31	-															84.48	4.98
5. Year 2 Average	.69	.18	.41	.80	-														86.49	3.96
6. Customized NBME Average	.79	.34	.29	.75	.74	-													75.75	5.69
7. CBSE	.76	.29	.15	.59	.56	.82	-												193.06	21.84
8. CBSSA Average	.80	.35	.25	.65	.68	.85	.79	-											212.87	18.54
LASSI Subscales																				
9. Anxiety	.26	.34	-.01	.05	.13	.31	.31	.33	-										65.95	26.26
10. Attitude	.04	-.29	-.03	.04	.01	-.00	.06	.09	.12	-									49.96	24.94
11. Concentration	.28	-.14	-.03	.16	.10	.12	.24	.17	.39	.42	-								53.05	27.51
12. Information Processing	-.05	.03	.18	.03	-.01	-.07	-.02	-.08	.09	.26	.28	-							56.54	26.80
13. Motivation	.12	-.15	.17	.18	.27	.11	.06	.11	.15	.31	.47	.38	-						64.00	22.61
14. Self-Testing	.14	-.14	.06	.26	.24	.17	.26	.16	-.01	.29	.34	.60	.36	-					56.45	28.10
15. Selecting Main Idea	.14	.19	.15	-.01	.08	.19	.27	.21	.53	.05	.44	.27	.34	.15	-				55.37	23.44
16. Study Aids	-.07	-.32	.12	.09	.01	-.07	-.01	-.12	-.29	.37	.11	.21	.07	.30	-.01	-			36.47	24.98
17. Time Management	.26	-.20	.03	.45	.31	.25	.29	.28	.03	.43	.48	.14	.49	.45	.16	.32	-		61.01	29.66
18. Test Taking Strategies	.17	.11	.25	.20	.24	.23	.22	.20	.52	.24	.51	.31	.52	.27	.62	.08	.43	-	61.84	23.44

r = ±.19 significant at p< .05; r = ±.26 significant at p< .01

### Regression model 2

The seventeen independent variables accounted for 71.4% of the variance in Step 1 performance (Table 3). The two statistically significant predictors were CBSSA (β = .338) and Concentration (β = .254) (Table 3).

Concentration (Model 1 - β = .264; Model 2 - β = .254) is the only study strategy correlated with Step 1 performance. The other statistically significant predictors are: Model 1 - Customized NBME Average (β = .315) and Year 2 Average (β = .280); Model 2 - CBSSA Average (β = .338). 

## Discussion

The purpose of this study was to examine the relationship between learning and study skills and USMLE Step 1 performance. The results of this study indicate that concentration is the only study strategy that appears to be predictive of USMLE Step 1 performance. This finding is consistent with the finding that concentration was significantly associated with National Board of Chiropractic Examiners (NBCE) performance levels. ^18^ This finding is also consistent with Nideffer^22^ and Maynard’s^24^ claims that concentration is “central to performance” and that “concentration is essential for performing one’s best.”^25^ The implications are that basic skills related to self-regulation geared toward enhancing study skills, personal factors, and task management can all contribute to the improvement of concentration and must be considered when designing educational interventions and working with students on exam preparation.^26^

What, then, are some tasks to offer students opportunities to self-regulate their ability to concentrate? Helping students find ways to control their stress levels, establish the appropriate physical environment, and use strategies to help them get focused and stay focused while they study would help them with concentration. Teaching them learning and study strategies such as being an active learner, using goal-setting techniques, and time management would also be beneficial.^27^

In terms of curriculum planning, there are techniques that faculty can implement in their teaching to help improve student concentration.^26,27-29^ Chunking lecture content and utilizing the pause procedure are two simple techniques which do not require a lot of time on the part of the faculty and can make a significant difference.^28^ Chunking is simply a practice of breaking up lecture content. Instead of a one hour lecture, there are four mini-lectures with three intervals of short duration which may be student interaction, a quiz, a written assignment, or an opportunity to interact with other students in some format. Williamson & Schell^24^ demonstrated in multiple studies^30-35^ how this method helped to hold students’ attention. Ruhl & Suritsky,^31^ indicated students who were instructed using the pause procedure, two minute pauses every twelve to eighteen minutes during the lecture,^36^ could recall more facts, vocabulary and ideas from lectures immediately following instruction than students presented with an entire lecture at once. They also outperformed the other group on an exam given one week later.^35^

**Table 2.** Model 1 Summary (n=79) ^[Table-fn fn15891]^

Variable	*β*	*t*	*(p)*
MCAT	-.018	-.228	.820
UGPA	-.066	-.941	.349
Year 1 Average	.155	1.196	.235
Year 2 Average	.280	2.358	.021
CBSE	.315	2.826	.006
Customized NBME Average	.248	1.764	.082
Anxiety	-.041	-.454	.651
Attitude	-.060	-.756	.452
Concentration	.264	3.005	.004
Information Processing	.083	.959	.341
Motivation	-.059	-.664	.509
Self-Testing	-.138	-1.432	.156
Selecting Main Idea	-.027	-.300	.765
Study Aids	-.049	-.634	.528
Time Management	-.047	-.470	.639
Test Taking Strategies	-.073	-.743	.459

Model 1: [R=.846, R2=.716, F=12.419, df =16, p <.01]

**Table 3.** Model 2 Summary (n=79) ^[Table-fn fn15892]^

Variable	*β*	*t*	*(p)*
MCAT	.005	.065	.949
UGPA	-.065	-.871	.387
Year 1 Average	.179	1.330	.188
Year 2 Average	.192	1.533	.130
CBSE	.247	1.973	.053
Customized NBME Average	.091	.566	.573
CBSSA	.338	2.562	.013
Anxiety	-.030	-.313	.755
Attitude	-.050	-.615	.541
Concentration	.254	2.856	.006
Information Processing	.069	.723	.472
Motivation	-.013	-.150	.882
Self-Testing	-.144	-.1.514	.135
Selecting Main Idea	-.066	-.713	.478
Study Aids	.010	.128	.898
Time Management	-.070	-.689	.493
Test Taking Strategies	-.046	-.456	.650

Model 2: [R= .881, R2 =.776, F= 12.452, df =17, p <.01]

Providing individualized training to help students improve concentration skills should be implemented. For example, one-on-one training utilizing q-bank questions may be beneficial. When a student reaches the point that his/her attention is about to wane, the instructor could encourage him/her to do five more questions and/or spend five more minutes on the question answering task. After doing several sessions utilizing this practice, the student will likely extend his/her concentration time and will also begin to practice this strategy to increase concentration time. 

Since the CBSE and CBSSA averages are also statistically significant predictors of USMLE Step 1 performance, these assessments, in addition to the LASSI, may be utilized in a variety of ways. For example, the CBSE may be administered on two occasions. The first administration may be used for baseline data along with the LASSI pre-test. Then, structured concentration training and coaching described above may be provided. The CBSE may then be administered again along with the LASSI as post-tests. This would enable one to determine if the intervention was successful in approving CBSE and/or study strategies measured by the LASSI. The CBSSA assessments should also be incorporated as benchmarks during the 4-6 weeks of the intense study period prior to the exam. Since concentration is the only study strategy identified as correlated with Step 1 performance, individualized test taking support designed to improve concentration may be utilized accordingly. Teaching students to practice and utilize certain techniques aimed at improving concentration skills when preparing and taking exams may result in higher Step 1 scores.

### Limitations

Limitations of this study include reliance on the LASSI, which is a self-report measure to determine students’ learning and study strategies. Even though the items are not presented in a manner that would influence participants to answer the way the investigator would like them to answer, the data may not accurately represent the actual study strategies that students utilized. Another limitation is the possibility that findings are due to the unique characteristics of the sample of first year medical student participants. One cohort was included in this study, so it limits the generalizability of the findings. This study should be replicated to determine if concentration is correlated with Step 1 scores in other cohorts. Furthermore, the decision to examine subscales in order to explore and suggest interventions instead of examining factors (will, skill, and self-regulation) may have yielded interpretation challenges. The will, skill, and self-regulation factors should also be examined, in addition to the ten study strategy subscales. Future researchers may also consider investigating if there is a relationship between self-directed learning readiness, as measured by the Self Directed Learning Readiness Scale (SDLRS) or another scale, and USMLE Step 1 exam performance. Investigating study strategies utilized by students that scored poorly on the USMLE Step 1 exam should also be examined.

Even if the data does not yield the exact findings noted in this study, utilizing the LASSI study strategy assessment appears to be beneficial. The instrument is user-friendly, economical, electronically administered and may be used to quickly identify those who need help improving certain skills such as concentration. Therefore, its’ incorporation is suggested. Providing individualized training sessions which encourage self-evaluation, reflection, ^37^ and techniques to improve study skills identified by the LASSI performance profiles may also improve academic and standardized test performance.
